# The effects of shade on cattle mobility, behavior, and dark cutting during lairage at a commercial slaughter facility in the United States

**DOI:** 10.1093/jas/skag018

**Published:** 2026-01-27

**Authors:** Lauren Dean, Paxton Sullivan, Lacey Alexander, Robert J Delmore, Lily Edwards-Callaway

**Affiliations:** Department of Animal Sciences, Colorado State University, Fort Collins, CO 80523, United States; Department of Animal Sciences, Colorado State University, Fort Collins, CO 80523, United States; Cargill Protein Headquarters, 825 E Douglas Ave, Wichita, KS 67202, United States; Department of Animal Sciences, Colorado State University, Fort Collins, CO 80523, United States; Department of Animal Sciences, Colorado State University, Fort Collins, CO 80523, United States

**Keywords:** cattle comfort, heat mitigation, lairage, meat quality, survey, welfare

## Abstract

Time at the slaughter plant during the preslaughter phase is a critical period in livestock production. Although time spent in this phase is relatively short, it can significantly impact cattle comfort and welfare. The purpose of this study was to determine the effect of shade during lairage on cattle mobility, behavior, and dark cutting at a commercial slaughter facility. Two shaded (148.6 m^2^ of 70% shade cloth) and two non-shaded pens were treatment pens. One hundred forty-two groups of cattle were included in the study between May and October 2023. Cattle mobility was scored using the Meat Institute’s four-point scale (1 = normal to 4 = extremely reluctant to move) as animals both entered and exited their lairage pen. Video footage of cattle during lairage was evaluated using instantaneous scan sampling with a 5-minute interval to quantify lying, standing, drinking, and motion. The frequency of mounting was also evaluated using behavior sampling during the entire lairage period. Treatment, time in lairage, and space allowance were predictor variables for all behavior outcomes; treatment, sex, weight, space allowance, and temperature humidity index (THI) were predictor variables for dark cutting. There was no impact of shade on behavior and quality outcomes (*P* > 0.05). An increase in lairage duration was associated with an increased odds of cattle lying down (OR: 1.0064, 95% CI: 1.0057, 1.0071, *P* < 0.0001) and decreased odds of cattle motion (OR: 0.9937, 95% CI: 0.9889, 0.9979, *P* = 0.0057). An increase in space allowance was associated with an increased odds of cattle lying down (OR: 1.9112, 95% CI: 1.6012, 2.2693, *P* < 0.0001), decreased odds of cattle motion (OR: 0.14, 95% CI: 0.03, 0.48, *P* = 0.0078), and increased odds of dark cutting (OR: 2.51, 95% CI: 1.80, 3.44, *P* < 0.0001). An increase of THI was associated with decreased odds of dark cutting (OR: 0.96, 95% CI: 0.92, 0.99, *P* = 0.0189). All steer pen groups were associated with decreased odds of dark cutting (OR: 0.39, 95% CI: 0.22, 0.70, *P* = 0.0017). A survey was also conducted with plant employees to provide insight to their perspectives on the use of shade in lairage (*n* = 13). The majority (84.6%) of employees agreed that shade should be used in holding pens. While shade was not found to impact measured outcomes, this study provided important insight on how lairage environments influence cattle welfare.

## Teaser

The provision of shade in commercial slaughter facilities was not found to impact how cattle spent their time in holding pens or the prevalence of normal mobility and dark cutting, however, space allowance and time in lairage were associated with measured outcomes.

## Introduction

The importance of animal welfare in the beef industry is reflected in heightened consumer concern, retailer expectations and producer animal care programs, and thus is an integral component of the cattle industry (Wigham et al. 2018; Edwards-Callaway and alvo-Lorenzo 2020). Over the past several decades consumers have consistently been interested in how livestock are treated, and studies continue to demonstrate how welfare claims can influence consumer decisions to purchase animal-based proteins worldwide (Alonso et al. 2020; FMI 2025). While the preslaughter period can be stressful due to travel, increased handling and new environments, the industry has strived to continuously mitigate these stressors. Adherence to federal regulations in the United States (Humane Slaughter Act 1978; Twenty-Eight Hour Law 1994) and implementation of best management practices (BQAT 2020; Meat Institute 2021; BQA 2025) throughout the beef industry promote the well-being of livestock during the pre-slaughter period. Animal handling and facility provisions during lairage directly impact animal welfare and can decrease the impact of stressors during the preslaughter period (Cockram and Corley 1991; Grandin 2000; Grandin 2018; Wigham et al. 2018; Edwards-Callaway and Calvo-Lorenzo 2020; Grandin 2020).

Heat stress, a known welfare concern within all sectors of the cattle industry, is particularly important when evaluating cattle comfort and welfare (Polsky and Von Keyserlingk 2017; Brown-Brandl 2018; Lees et al. 2019; Edwards-Callaway et al. 2021). During heat events, cattle performance and behaviors can change to cope with heat stress (Edwards-Callaway et al. 2021). Meneses et al. (2021) explored cattle behaviors in feedlots and indicated that behavioral patterns are a way to understand animals’ ability to (or failure to) cope with their environment. In the feedlot sector, the addition of shade was associated with increases in dry matter intake and average daily gains (Mitlöhner et al. 2001; Mitlöhner et al. 2002), decreased death loss (Busby and Loy 1997) and reduced the incidence of dark cutters (Mitlöhner et al. 2002) in comparison to non-shaded cattle. Mitlöhner et al. (2002) also reported an increase of lying down in shaded heifers compared to non-shaded heifers. Despite the positive impacts heat mitigation has on feedlot cattle, there is minimal knowledge on heat mitigation in slaughter facilities during lairage. To the authors’ knowledge only one study has explored heat mitigation in lairage facilities and found sprinklers to be the most common approach, followed by shade and fans (Davis et al. 2022). Additionally, research has shown that cattle mobility was impacted by temperature and THI during transportation and at the slaughter facility (González et al. 2012; Mijares et al. 2021), however, the impact of shade on mobility at the plant has not been explored.

Postmortem indicators can also be used to evaluate the impact of lairage on cattle. Dark cutting beef is a meat quality defect caused by inadequate pH decline postmortem resulting from preslaughter stress depleting glycogen stores (Scanga et al. 1998; Ponnampalam et al. 2017). A multitude of factors can influence the occurrence of dark cutting including weather, transportation, stocking density or time in lairage (Ponnampalam et al. 2017). Steel et al. (2022) explored heat load prior to slaughter, finding sudden changes in climate two days prior to slaughter were associated with and increased frequency of dark cutting. Providing shade at the feedlot was found to decrease the occurrence of dark cutting in fed cattle (Mitlöhner et al. 2002). Heat mitigation in lairage and its impact on dark cutting frequency, however, has been underrepresented in the literature.

Past research, although primarily conducted in feedlots, has provided insight as to how extreme weather events can alter behavior and meat quality of cattle. Therefore, the objective of this study was to assess the impact of shade at a slaughter facility and its effect on fed cattle mobility, behavior, and incidence of dark cutters.

## Materials and methods

This project included a live animal component and an employee survey. All animal procedures conducted during this research were observational, and therefore the project was reviewed and deemed exempt by the Colorado State University Institutional Animal Care and Use Committee (IACUC #4201). The employee survey was reviewed and deemed exempt by the Institutional Research Board (IRB #4544).

### Cattle and facility description

Data was collected at one USDA inspected slaughter facility in the western region of the United States; the facility processed approximately 4,000 cattle per day over two 8-hour shifts. The study was conducted between May 2023 and October 2023, resulting in 31 collection days. Collectively, one hundred and forty-two pen groups (12,736 total cattle) were included in the study: 60 shaded pen groups (*n* = 4,393 cattle) and 82 control pen groups (*n* = 8,343 cattle). Observations were made during both slaughter shifts, as well as some groups of cattle that arrived the night prior to their slaughter day (i.e., overnight cattle). Data collection occurred antemortem during unloading, lairage, and movement to the slaughter floor. Upon arrival at the plant, cattle were identified with a lot (i.e., contemporary group from the feedyard) number and allocated to lairage pens based on lot size; some lots were separated into different pens. A lairage pen of animals was referred to as a pen group and the pen group was the experimental unit. Cattle in the study came directly from feedlots, were primarily under 30 months of age, both steers and heifers, and primarily crossbred Angus with some lots having Bos indicus influence. The following information was collected from each pen group: number of animals in the lot, location of origin (i.e., location of the producer that shipped the cattle), average plant live weight (calculated by dividing the total truck weight by the total number of animals on the trailer), hide color (>75% of the animals in the group had a black hide, 75–25% of the animals in the group had a black hide, or <25% of the animals in the group had a black hide), and sex (steer, heifer, or mixed steer and heifer group). Space allowance per pen group was calculated by dividing the total area of the lairage pen by the total number of animals in the pen for each pen group. Lairage duration was defined as the time the last sub-group entered the holding pen and the gate was closed to the time when the first sub-group was removed from the holding pen to be moved to slaughter; note that lairage duration was not controlled during the study and was determined by plant scheduling. Google Maps (Google, Mountain View, California) was utilized to determine the distance traveled from the location of origin directly to the slaughter facility; if multiple routes were given, the shortest distance was chosen. The authors acknowledge this may not reflect the exact distance traveled.

### Treatments

There were two treatments in this study: shade in holding pens (shade) and no shade in holding pens (control). A total of four pens were designated as research pens at the facility: two shaded pens (each 148.64 m^2^; 72 head capacity per pen) and two control pens (202.71 and 222.97 m^2^; 99 and 109 head capacity, respectively). The pens with shade were selected by plant personnel due to their similar size. The shade structures (Strobel Manufacturing, Inc., Clarks, Nebraska) were 4.45 m at the highest center point and sloped down to a height of 3.66 m along the perimeter. The structure used a 70% mesh Shur-Co^®^ Tarp (Shur-Co^®^, Yankton, South Dakota) covering 12.19 × 12.19 m that provided 148.64 m^2^ of shade, shading the two designated shade pens. The base of the structure was in one of the two shaded pens, reducing the square meters of one shaded pen to 145.67 m^2^ and the capacity to 71 head. This installation was the only shade in the facility other than immediately before slaughter at the entrance to the serpentine, which all cattle were exposed to; a minimal amount of time was spent in these areas. Flooring in all study pens was stamped concrete.

### Environmental measurements

Ambient temperature (T), humidity (transformed to relative humidity, RH = humidity/100), and ground temperature were collected. Ambient temperature and humidity were recorded approximately every 30 min on each data collection day using weather sensors (HTP.xw, SensorPush, Brooklyn, New York). One sensor was attached to a camera extender pole (DocaPole, Memphis, Tennessee) near the unshaded pens, and the other was attached under the shade structure. A temperature and humidity reading was taken approximately every 30 min during the pen group’s total lairage duration. Temperature-humidity index (THI) was then calculated: THI = 0.8 * T + RH (T − 14.4) + 46.4. The calculated THI was then classified into categories: Normal <75, Alert 75–78, Danger 79–83, or Emergency >84 (Eirich and Woolsoncroft 2018). Ground temperature of each treatment was recorded approximately once per hour using a handheld infrared thermometer (IR10, Klein Tools, Illinois, United States) in four locations per treatment, taken only on one side of the pens due to cattle being present and safety as a priority. The four temperatures were then averaged for one reading per hour. An average was calculated for the final environmental recordings (i.e., THI or ground temperature) of each pen group.

### Mobility

Video equipment (Hero 5 and Hero 10, GoPro, San Mateo, California) was used to capture cattle in motion at two different time points for each pen group: 1) after unloading as they were being moved to their holding pen (pre-lairage) and 2) at the end of lairage when cattle were moved from their holding pen to the slaughter floor (post-lairage). Cattle were moved into lairage pens in smaller groups (approximately 40 cattle) and moved out of lairage pens to slaughter in subgroups. Pre-lairage mobility cameras were placed just past the unloading pens to capture movement down the alley to their lairage pens and provided an elevated side view of each animal. Post-lairage mobility cameras were placed just past the final treatment pen to capture movement down the alley to slaughter and provided an elevated front view of each animal. One hundred and twenty-four pen groups (*n* = 50 shaded pen groups, *n* = 74 control pen groups) were assessed for mobility. Two observers with extensive experience evaluating in-plant cattle mobility scored mobility using the video footage. A four-point scale (Table 1, Meat Institute 2015) was used to score animals in the study at both time points. The number of animals within each of the four mobility categories was tallied per pen group.

**Table 1:** North America Meat Institute (Meat Institute 2015) mobility scoring system for cattle.

Mobility score	Definition
**1**	Normal, walks easily, no apparent lameness, no change in gait
**2**	Exhibits minor stiffness, shortness of stride, slight limp, keeps up with normal cattle
**3**	Exhibits obvious stiffness, difficulty taking steps, obvious limp and discomfort, lags behind normal cattle
**4**	Extremely reluctant to move even when encouraged, statue-like

### Pen behavior

Cattle were video recorded during the duration of lairage. Cameras (Hero 5 GoPro, San Mateo, California) were attached to extender poles (DocaPole, Memphis, Tennessee) and secured to existing fences around the target lairage pens approximately 2.44 meters away from the entrance of treatment pens. The poles were extended approximately 7.62 meters high over the control pens, providing a top view of all animals in the pens, and approximately 3.66 meters high over the shaded pens, providing an angled top view of all animals in the pens, to capture a full view of the pens. Cameras were positioned to capture two entire lairage pens (i.e., there was one camera capturing both shaded pens and one camera capturing both control pens). One hundred eight pen groups (*n* = 55 shaded pen groups, *n* = 53 control pen groups) were assessed for the following behaviors: lying, standing, water, motion, and mounting. Videos were viewed at a later date to record cattle behaviors as defined in Table 2. Behavior sampling was performed for the entire lairage duration to count the number of times mounting occurred. Instantaneous scan sampling was used to quantify the number of animals drinking water, lying, and standing using 5-minute intervals (Bateson and Martin 2021), beginning 5 min after lairage began. During each scan, all cattle in the pen group were observed and counted as performing one of three behaviors to provide a summed number of animals performing each behavior at each interval. Drinking was counted both when animals were drinking water and if their head was over the trough. A motion index quantified animals in motion (e.g., movement such as walking or shifting weight) and was scored using a three-point scale: 1) less than 1/3 of cattle moving, 2) 1/3 to 2/3 of cattle moving, 3) more than 2/3 of the cattle moving at each interval scan.

**Table 2:** Definitions of measured behaviors during lairage.

Behavior	Definition
**Lying^1^**	Animal is recumbent, not supported by legs
**Standing**	In upright position supported by their legs; can be walking or standing still
**Drinking^1^**	Animal has head in or over water trough
**Motion**	Weight shifting or walking; 1: <1/3 of the cattle moving; 2: 1/3–2/3 moving; 3: >2/3 moving
**Mounting^1^**	Animal positions body on top of another animal’s topline

1 Adapted from Meneses et al. (2021).

### Dark cutters

Quality data was collected from 136 study pen groups (*n* = 56 shaded pen groups, *n* = 80 control pen groups). Carcass data for each pen group was obtained from the plant records. An E + V camera was used to sort normal, off-color, and dark-color carcasses. The following scale was used to categorize carcasses as a predictive measure of L* color that is used for in house lean color determination: normal >59, off color 56–59 and dark cutting <55. Off-color is termed for beef not recognized as dark cutting but is still undesirable, resulting in the downgrading of a carcass. The number of each category per pen group was summed.

### Survey

In August 2023, approximately three months after the study began, an employee survey was conducted. The survey was optional, and no incentive was provided. It was available in both English and Spanish, and employees were able to complete it on their own or have it read to them and provide answers verbally. All employees involved in daily handling of cattle on both shifts were asked to participate; this included hourly employees, supervisors, and managers. The survey included four Likert scale questions, and each question had a follow-up free response question to explain their answer. A final question asked how many years the ­employee had worked with cattle in holding pens.

### Statistical analysis

The pen group served as the experimental unit for all analysis. Pen groups were excluded if any subgroups entered or left the pen more than 30 min apart from each other. Incomplete data for some parameters (i.e., missing post-lairage mobility score or missing space allowance information) resulted in a discrepancy of sample sizes between analyses due to the exclusion of pen groups for some outcomes (i.e., dark cutting or mobility). Analysis was performed in R Statistical Software (v4.4.2; R Core Team 2022). Descriptive statistics were calculated for all variables considered. Due to the low occurrence of mobility scores 3 and 4, mobility was collapsed into two categories: normal (score of 1) or abnormal (score of 2, 3, or 4). Only pen groups that had pre-lairage and post-lairage mobility scores were included for the mobility analysis. The number of animals per the two mobility categories (i.e., normal and abnormal) were summed pre- and post-lairage for each pen group. Meat quality data was also collapsed into two categories (i.e., normal or dark/off color) and summed per pen group. The five-minute behavior scans during lairage were averaged to get each pen group’s average of cattle drinking water and standing or lying during lairage. Once the mode was calculated for motion index scores, the third category (>2/3 of cattle moving) was no longer included, and motion was reported as 0: <1/3 of the cattle moving or 1: >1/3 of the cattle moving. Mounting was reported as a rate and calculated by dividing the total number of mounting events observed by the product of the total number of animals in the pen and the total duration time.

Responses to the survey questions were entered into Microsoft Excel (Microsoft, Redmond, WA). Summary statistics were performed on the survey Likert questions. Due to the small sample size, formal qualitative analysis was not conducted on the free responses.

All models, except mounting and motion, utilized a grouped binary logistic regression approach to count successes and failures within each pen group; this approach models the likelihood of events (i.e., behavior, normal mobility, or dark cutting) per pen group by adjusting for the number of possible outcomes (i.e., total head in the pen group). Post-lairage mobility analysis was completed using a mixed model (package: glmmTMB, v: 1.1.10) by using the assigned unique identifier to each pen group (i.e., 1, 2, 3, etc.) as the random effect and included treatment, time in lairage, and space allowance as predictor variables. All behavior analysis included treatment, time in lairage, and space allowance as predictor variables. Mounting behavior was analyzed using a negative binomial generalized linear model. A generalized linear model was used for motion analysis.

An Akaike information criterion (AIC) (package: MuMIn, v: 1.48.4) was used to evaluate the dark cutting regression model. Treatment, sex, weight, space allowance, temperature-humidity index, hide color, distance traveled, and lairage duration were considered as predictor variables; based on subject matter knowledge, treatment, sex, and weight were forced to stay in the model. A full model was fit with all predictor variables, and an automated model selection was applied. The lowest AIC was selected and included treatment, sex, weight, space allowance, and temperature-humidity index in a generalized linear model.

Type II tests were used to analyze models; a *P* ≤ 0.05 was deemed as statistically significant, and 0.05 ≤ *P* ≤ 0.10 signified a trend.

## Results

### Cattle and facility description

Cattle were placed in lairage pens based on plant logistics, resulting in the difference between shaded and control pens. All steer pen groups were most frequent (*n* = 57, 40.14%), followed by mixed pens of heifers and steers (*n* = 53, 37.32%), and all heifer pens (*n* = 32, 22.54%). Overall, more than half of the pen groups consisted of >75% of the cattle having black hides (*n* = 101, 71.13%). Pens with 25 to 75% black-hided cattle (*n* = 33, 23.24%) and pens with less than 25% black-hided cattle (*n* = 8, 5.63%) made up the remaining population. The average live weight ranged from 489.88 to 721.21 kg with a mean of 632.52 ± 49.3 kg (mean ± SD). Lairage duration ranged from 54 to 806 min with an average of 325.25 ± 207.31 min. On average, the space allowance per animal in this study was 2.07  ± 0.46 m^2^ with a range of 1.61 to 4.21 m^2^. The average distance traveled was 279.59 ± 240.88 km, ranging from 6.76 to 825.59 km.

### Environmental measurements

Table 3 reports ambient temperature, humidity, and ground temperature for the shaded and control pens. Additionally, THI is reported, including a summary of how many pen groups fell within each THI category. The majority of shaded pens (*n* = 23) fell in the *Normal* THI category, while the THI category *Danger* was most prominent in control pens (*n* = 21). All parameters numerically varied between treatments. The largest numerical difference was seen in maximum ground temperature between the Shade and the Control pens, 26.14 and 36.42 °C, respectively.

**Table 3:** Environmental measurements (ambient temperature^1^, humidity, ground temperature,^2^ and THI^3^) taken during lairage in the Shade and Control pens at the slaughter facility.

Variable	Shade	*n*	Control	*n*
**Ambient temperature, °C**		47		49
** Minimum**	15.87		15.00	
** Mean ± SD**	28.25 ± 5.22		30.66 ± 8.14	
** Maximum**	36.28		43.09	
**Humidity**		47		49
** Minimum**	19.83		16.70	
** Mean ± SD**	45.92 ± 20.57		38.59 ± 22.51	
** Maximum**	92.17		99.45	
**Ground temperature, °C**		37		38
** Minimum**	14.10		17.50	
** Mean ± SD**	22.72 ± 2.99		25.48 ± 4.45	
** Maximum**	26.14		36.42	
**THI**		47		49
** Minimum**	60.45		58.99	
** Mean ± SD**	74.47 ± 5.11		75.65 ± 7.85	
** Maximum**	80.17		87.06	
** Normal**		23		19
** Alert**		14		5
** Danger**		10		21
** Emergency**		0		4

1 Ambient temperature and humidity were collected approximately every 30 min.

2 Ground temperatures were collected approximately once per hour.

3 THI was calculated using THI = 0.8 * T + RH (T − 14.4) + 46.4 and categories (i.e., normal) followed the chart from Eirich and Woolsoncroft (2018). The values in the n columns represent the number of pen groups falling within each of the listed THI categories (normal, alert, danger, and emergency).

### Mobility

Table 4 provides a summary of pre-lairage and post-lairage mobility scores by treatment. Treatment, space allowance, and time in lairage were not found to have an association with mobility scores (*P* > 0.10), although pre-lairage mobility had a tendency to be associated with mobility post-lairage (*P* = 0.06, Table 5).

**Table 4:** Frequency of fed cattle mobility score pre-lairage and post-lairage with or without shade (*n* = 11,109).

Mobility	Shade n, %	Control n, %
Normal score^1^		
Pre-lairage	2880, 79.34	6003, 80.26
Post-lairage	2769, 76.28	6249, 83.55
Abnormal score^1^		
Pre-lairage	750, 20.66	1476, 19.74
Post-lairage	861, 23.72	1230, 16.45

1 Mobility was scored as either: 1 = normal, walks easily, no apparent lameness, no change in gait; 2 = minor stiffness, shortness of stride, slight limp, keeps up with normal cattle; 3 = obvious stiffness, difficulty taking steps, obvious limp, obvious discomfort, lags behind normal cattle; 4 = extremely reluctant to move even when encourages, statue like. Normal was considered score 1 and abnormal was scores of 2, 3, and 4. Pre-lairage = mobility score upon unloading as they exited the unloading pen. Post-lairage = mobility score upon movement to the stunning area as they exited the holding pen at the end of lairage

**Table 5:** Mobility score^1^ grouped binary logistic regression analysis of fed cattle (*n* = 124 pen groups) housed in lairage pens at a slaughter plant with or without shade.

Variable	Estimate	SE	*P*-value	OR (95% CI)^2^
Treatment				
Control	*Referent*	–	–	–
Shade	−0.231	0.161	0.2550	0.793 (0.533,1.182)
Mobility (pre-lairage)	0.009	0.005	0.0667	1.009 (0.999, 1.019)
Space ­allowance, m^2^/animal	0.062	0.222	0.7679	1.064 (0.706, 1.603)
Time in ­lairage (min)	−0.0001	0.0003	0.8854	0.999 (0.999, 1.001)

1 Mobility was scored as either: 1 = normal, walks easily, no apparent lameness, no change in gait; 2 = minor stiffness, shortness of stride, slight limp, keeps up with normal cattle; 3 = obvious stiffness, difficulty taking steps, obvious limp, obvious discomfort, lags behind normal cattle; 4 = extremely reluctant to move even when encourages, statue like. Normal was considered score 1 and abnormal was scores of 2, 3 and 4. Pre-lairage = mobility score upon unloading as they exited the ­unloading pen. Post-lairage = mobility score upon movement to the stunning area as they exited the holding pen at the end of lairage

2 An odds ratio >1 indicates that the variable is associated with a multiplicative increase in the odds of an animal performing a certain behavior, whereas an odd ratio <1 indicates that the variable is associated with a multiplicative decrease in the odds of an animal performing a certain behavior.

### Pen behavior

All in-pen behavior descriptive summaries by treatment are provided in Table 6. On average, cattle were typically observed standing in both shade (92.08 ± 5.54%) and control (91.37 ± 7.95%) pens.

**Table 6:** Percentage of animals performing behaviors (standing, lying, drinking, motion, and mounting) in pens groups with (*n* = 55 pen groups) or without shade (*n* = 53 pen groups).

Variable	Shade	Control
**Standing^1^, % animals**		
** Minimum**	73.41	61.45
** Mean ± SD**	92.08 ± 5.54	91.37 ± 7.95
** Maximum**	98.46	99.07
**Lying^1^, % animals**		
** Minimum**	0	0
** Mean ± SD**	3.59 ± 5.83	4.70 ± 8.07
** Maximum**	25	36.36
**Drinking^1^, % animals**		
** Minimum**	1.54	0.93
** Mean ± SD**	4.33 ± 1.31	3.93 ± 1.56
** Maximum**	6.91	7.89
**Motion^2^**		
** <1/3 cattle moving**	54.55	52.83
** >1/3 cattle moving**	45.45	47.17
**Mounting^3^**	10.62	11.38

1 Standing, lying, and drinking were scored as mutually exclusive actions.

2 Motion evaluated using the following scale: 0: <1/3 cattle moving; 1: >1/3 cattle moving

3 All instances of mounting that occurred within a pen group during the lairage period were recorded, and the rate of mounting was calculated by dividing the total instances of mounting by the product of the total animals in the pen and the lairage duration multiplied by 100.

No association was found between treatment and standing (*P* > 0.10) or lying (*P* > 0.10; Table 7). Lairage duration and space allowance impacted standing (*P* < 0.0001) and lying behavior (*P* < 0.0001; Table 7). The odds of cattle standing decreased with a one-unit increase of lairage duration (OR: 0.9964, 95% CI: 0.9959, 0.9970, *P* ≤ 0.0001) and space allowance (OR: 0.6433, 95% CI: 0.5600, 0.7419, *P* ≤ 0.0001). Comparatively, the odds of cattle lying increased with a one-unit increase of lairage duration (OR: 1.0064, 95% CI: 1.0057, 1.0071, *P* ≤ 0.0001) and space allowance (OR: 1.9112, 95% CI: 1.6012, 2.2693, *P* ≤ 0.0001). There was no evidence of an association between the amount of cattle drinking water and treatment (*P* > 0.10) or space allowance (*P* > 0.10; Table 7). The odds of cattle drinking water decreased with a one-unit increase of time in lairage (OR: 0.9990, 95% CI: 0.9981, 0.9999, *P* = 0.0469). There was no association found between treatment and the quantity of cattle being in motion (*P* > 0.10; Table 7). The odds of more cattle being in motion decreased with an increase in unit of space allowance (OR: 0.14, 95% CI: 0.03, 0.48, *P* = 0.0078). Similarly, a unit increase of lairage duration decreased the odds of more cattle being in motion (OR: 0.9937, 95% CI: 0.9889, 0.9979, *P* = 0.0057). Treatment, space allowance, and time in lairage were not found to have an association with mounting (*P* > 0.10; Table 7).

**Table 7:** Logistic regression of behavior^1^ performed by fed cattle in holding pens (*n* = 108) with or without shade during lairage.

Variable	Estimate	SE	*P*-value	OR (95% CI)^2^
**Standing**				
** Treatment**				
** Control**	*Referent*	–	–	–
** Shade**	−0.0136	0.0790	0.863	0.9865 (0.8452, 1.1522)
** Space allowance, m^2^/animal**	−0.4412	0.7169	<0.0001	0.6433 (0.5600, 0.7419)
** Time in lairage (min)**	−0.0035	0.0003	<0.0001	0.9965 (0.9959, 0.9970)
**Lying**				
** Treatment**				
** Control**	*Referent*	–	–	–
** Shade**	0.0114	0.1165	0.922	1.0114 (0.8042, 1.2703)
** Space allowance, m^2^/animal**	0.6478	0.0889	<0.0001	1.9112 (1.6012, 2.2693)
** Time in lairage (min)**	0.0064	0.0004	<0.0001	1.0064 (1.0057, 1.0071)
**Drinking water**				
** Treatment**				
** Control**	*Referent*	–	–	–
** Shade**	0.0929	0.1047	0.375	1.0973 (0.8929, 1.3465)
** Space allowance, m^2^/animal**	−0.0051	0.1269	0.968	0.9949 (0.7669, 1.2629)
** Time in lairage (min)**	−0.0009	0.0005	0.047	0.9990 (0.9981, 0.9999)
**Motion**				
** Treatment**				
** Control**	*Referent*	–	–	–
** Shade**	−0.0331	0.4288	0.9384	0.9674 (0.4167, 2.2561)
** Space allowance, m^2^/animal**	−1.9814	0.7453	0.0078	0.1379 (0.0255, 0.4834)
** Time in lairage (min)**	−0.0063	0.0023	0.0057	0.9937 (0.9889, 0.9979)
**Mounting**				
** Treatment**				
** Control**	*Referent*	–	–	–
** Shade**	0.0753	0.3739	0.840	1.0783 (0.4919, 2.3275)
** Space allowance, m^2^/animal**	0.2811	0.3674	0.444	1.3246 (0.7029, 2.8270)
** Time in lairage (min)**	0.0016	0.0016	0.312	1.0016 (0.9980, 1.0055)

1 Standing, lying, and drinking water were recorded as mutually exclusive events. Motion was defined as shifting weight between sides of the body and limbs or walking, and motion was assessed using the following binary scale: 0: <1/3 of the cattle were moving; 1: >1/3 of the cattle were moving.

2 An odds ratio >1 indicates that the variable is associated with a multiplicative increase in the odds of an animal performing a certain behavior, whereas an odd ratio <1 indicates that the variable is associated with a multiplicative decrease in the odds of an animal performing a certain behavior.

### Dark cutting

A total of 135 cattle, 1.1% of the study population, presented as dark cutting and off-color carcasses; 28.15% (*n* = 38) of this total were from the shade treatment, and 71.85% (*n* = 97) were from the control treatment (Table 8). No significant difference was detected between treatments (*P* > 0.10; Table 9). However, dark cutting was associated with multiple factors, including THI, space allowance, and sex (*P* < 0.05; Table 9). The odds of dark cutting and off-color carcasses were negatively associated with a unit increase of THI (OR: 0.96, 95% CI: 0.92, 0.99, *P* = 0.0189). Whereas the increase of space allowance increased the odds of cattle having dark or off-color carcasses (OR: 2.51, 95% CI: 1.80, 3.44, *P* < 0.0001). Pens with all steers had decreased odds of dark and off outcomes (OR: 0.39, 95% CI: 0.22, 0.70, *P* = 0.0017). No association was found between dark cutting and weight (*P* > 0.10; Table 9).

**Table 8:** Frequency of dark cutting and off color in fed cattle carcasses with or without shade during lairage.

Mobility	*N*	Shade (*n* = 3961)	Control (*n* = 8280)
		*n*, %	*n*, %
**Dark cutting**	31	7, 22.58	24, 77.42
**Off color**	104	31, 28.81	73, 70.19
**Dark + off**	135	38, 28.15	97, 71.85

*n*, individual cattle.

**Table 9:** Logistic regression of occurrence of dark cutting and off color^1^ in fed cattle carcasses with or without shade during lairage.

Variable	Estimate	SE	*P*-value	OR (95% CI)^3^
**Treatment**				
** Control**	*Referent*	–	–	–
** Shade**	−0.3417	0.2198	0.1199	0.71 (0.46, 1.08)
**THI^2^**	−0.0424	0.0181	0.0189	0.96 (0.92, 0.99)
**Space allowance, m^2^/animal**	0.9189	0.1638	<0.0001	2.51 (1.80, 3.44)
**Estimated weight, kg**	<0.0001	0.0012	0.9718	1.00 (0.99, 1.00)
**Sex**				
** Heifers**	*Referent*	–	–	–
** Mixed**	−0.5079	0.3017	0.0924	0.60 (0.33, 1.08)
** Steers**	−0.9421	0.2997	0.0017	0.39 (0.22, 0.70)

1 Dark cutting and off color carcasses were determined using a E + V camera with an in house predetermined scale. Dark cutting was <55 and off color was 56–59.

2 THI was calculated using THI = 0.8*T + RH (T − 14.4) + 46.4

3 An odd ratio >1 indicates that the variable is associated with a multiplicative increase in the odds of an animal performing a certain behavior, whereas an odd ratio <1 indicates that the variable is associated with a multiplicative decrease in the odds of an animal performing a certain behavior.

### Survey

Table 10 summarizes the survey results from 13 plant employees. More than half of the employees strongly agreed (*n* = 8) or agreed (*n* = 3) that “shade should be used in holding pens” and “prefer to work in the holding pens with shade” (*n* = 9). Employees who participated in the survey ranged in experience: a majority of participants (*n* = 6) worked in holding pens for 1 to 10 years, followed by those with less than one year of experience (*n* = 5) and two having more than 10 years of experience.

**Table 10:** Respondents were asked to select their level of agreement on a scale of strongly agree, agree, disagree, and strongly disagree with statements related to implementation of shade in the holding pens at the slaughter facility. There were a total of 13 employees included in the survey.

Statement	*n*			
	Strongly agree	Agree	Disagree	Strongly disagree
***Cattle are more difficult to handle when coming out of holding pens with shade compared to those without.***	1	2	6	4
***Cattle seem to be more uncomfortable in holding pens with shade compared to those without.***	0	2	4	7
***I prefer to work in holding pens with shade compared to those without.***	6	3	1	3
***Shade should be used in holding pens.***	8	3	1	1

## Discussion

While the impact that shade has on cattle behavior and meat quality has been explored in feedlots (Mitlöhner et al. 2001; Mitlöhner et al. 2002; Edwards-Callaway et al. 2021), there is limited research evaluating different heat mitigation techniques at slaughter. For example, Zhao et al. (2022) explored how showering cattle in lairage at a slaughter facility affected meat quality, but this study was conducted in cold weather. Although the provision of some type of heat mitigation is a standard among most slaughter facilities in the United States, the methods of implementation vary across facilities. Davis et al. (2022) found 81% of plants participating in their survey used sprinklers and 33% used a type of shade coverage to mitigate impacts from heat. The Davis et al. (2022) survey represented both plants that slaugthered fed cattle and those that slaughtered cull beef and dairy cattle. There was no impact of shade on the outcome variables in this study.

The cattle population characteristics in this study were representative of the current fed beef population slaughtered in the US (NBQA 2022), predominantly black-hided steers. On average, ­cattle weighed 632.52 kg, similar to the industry-reported average of 634.58 kg (USDA-NASS 2024).

### Mobility

Mobility scoring, which offers a way to visually assess physical animal condition, has been utilized to identify trends through audits such as the National Cattlemen’s Beef Association National Beef Quality Audit (NBQA; Eastwood et al. 2017) and is utilized as a key welfare indicator measured at slaughter (Meat Institute 2015; Edwards-Callaway et al. 2017). These benchmarking data indicate that many cattle arrive at commercial slaughter facilities with normal mobility (92%, Eastwood et al. 2017; 91.8%, Davis et al. 2024a). The current study reported 79.9% of cattle had normal mobility pre-lairage (79.34% Shade; 80.26% Control) and 81.18% post-lairage (76.28% Shade; 83.55% Control). Similar to the current study, others have reported normal mobility below the industry averages pre-lairage (74.6%, Mijares et al. 2021) and post-lairage (67.12%, Sullivan et al. 2024). Few studies have followed cattle through the slaughter facility to assess the impact of lairage on mobility changes (Hagenmaier et al. 2017a, 2017b), and this is an opportunity for future work.

While the impact of shade has been assessed on behavioral and physiological outcomes in feedlot cattle, to date, we were not able to find any studies that explored shade in relation to mobility scores at the feedlot or slaughter facility. Issues related to impaired mobility can often be caused by physical injuries (i.e., foot lesions, broken ligaments, etc.) but can also be impacted by other factors, which include transportation (González et al. 2012; Edwards-Callaway et al. 2017; Davis et al. 2024b), handling practices (Edwards-Callaway et al. 2017), and environmental conditions (Lee et al. 2018; Mijares et al. 2021; Davis et al. 2024b; Sullivan et al. 2024).

The current study reported a numerically lower THI in shaded pens (74.47) compared to control pens (75.56), yet no association between treatment and mobility scores post-lairage was found. Although the current study found that with the provision of shade the THI was numerically lower, the overall humidity was numerically greater (45.92 shade vs 38.59 control). Humidity increases the risk of heat stress in cattle even with the availability of shade. While shade blocks the direct sunlight, impacting how cattle dissipate heat (Edwards-Callaway et al. 2021), the rise in humidity that occurred during the current study could have altered the effect of the shade. It should be noted that in this study, shade availability also changed throughout the day in the treatment pens, which likely impacted the influence shade did or did not have on the study cattle. Optimal shade allowance has not been evaluated in lairage pens because shade in general has not been studied. In feedyards, the recommendation for total shade being provided per animal varies (da Silva and Maia 2012). Additionally, due to the size and density of lairage pens, likely most shade systems would attempt to provide full coverage of the holding area. A critical consideration for future work is the evaluation of the type of shade structure to consider different heights, orientations, and materials to maximize cattle comfort benefits and assess how different shade structures may change the microclimate of the pen. While no association was found in the current study, literature is limited in understanding the impact that time in lairage and space allowance during lairage have on mobility (Davis et al. 2024b; Sullivan et al. 2024) and is an area of opportunity for future work.

### Pen behavior

In any sector of the industry, creating environments that allow cattle to express natural behaviors is paramount. as the expression of highly motivated behaviors is important to overall animal welfare (Arave and Albright 1981; Napolitano et al. 2009). The absence of negative behaviors or occurrence of abnormal behaviors may also be good indicators of comfort and welfare during lairage (Phillips 2008; Relić et al. 2012; Meneses et al. 2021). Aggressive behaviors have been found to increase in feedlot cattle during times of heat stress due to frustration (Polsky and Von Keyserlingk 2017). The current study did not assess direct aggression (i.e., head butting) but did report mounting. As reviewed by Blackshaw et al. (1997), buller steer syndrome (i.e., animals being repeatedly mounted by others) is commonly seen in feedlots and may be caused by a multitude of factors such as social hierarchy, weather, and stress; this occurrence of excessive mounting is a welfare problem due to the negative effects it may have on that animal. While mounting was not found to be associated with treatment, time in lairage, or space allowance, it is important to note that although some pens had high counts of mounting observations, it was typically few animals that repeatedly performed this behavior, based on anecdotal observations. Future studies should explore other behaviors of frustration, such as head butting, to assess the impact of shade in lairage.

Lying and standing behaviors also provide insight as to how lairage impacts cattle comfort, as cattle are highly motivated to lie down, especially when deprived of lying for some time (Metz 1985; Jensen et al. 2004; Tucker et al. 2018). Previous studies found shade to be associated with an increase in lying behaviors in feedlot cattle (Mitlöhner et al. 2002; Sullivan et al. 2011); however, the current study found no association between lying or standing and treatment. Generally, there is limited space for animals to lie down in lairage pens during the day, and the space allowance is lower than in feedlot pens, which may explain some of the differences between studies. Although most study cattle were often observed standing (91.73%), an increase in lairage duration was associated with the odds of more cattle lying down in the current study. Similarly, Cockram (1990, 1991) analyzed behavior in lairage, comparing the origin of the animals, and reported that market cattle had an increase in lying behavior as time in lairage increased compared to cattle shipped directly from a farm; additionally, Cockram (1990, 1991) reported that the availability of bedding impacted lying behavior. Interestingly, these findings suggest lying behavior in lairage may be due to exhaustion of multiple transportation events and new environments (Cockram 1991). Cockram (1990) reported that cattle settled in lairage at around 3.5 h. The current study had an average of about 5 h in lairage, well over when cattle are found to settle, providing sufficient time for more cattle to exhibit lying behaviors.

Lying and standing behaviors were also found to be associated with space allowance during lairage, with an increase of space allowance being associated with a decrease in the amount of cattle standing and conversely, an increase of cattle lying. Most of the current literature surrounding lying behaviors have focused on dairy cattle (Hill et al. 2009; Lobeck-Luchterhand et al. 2015; Winckler et al. 2015). In a feedlot facility, Mayes et al. (2025) observed feedlot cattle and found similar results to the current study, that with an increase of pen space more steers were lying. The current study’s average space allowance (2.07 m^2^/animal) aligns with the Meat Institute’s guidelines for lairage space allowance (Meat Institute 2021). While the increase of space allowance allows more animals to lie down, government regulations only require space for cattle to lie down when they are being held overnight (CFR 1979). Even so, further understanding how the lairage environment may inhibit lying behavior is important when discussing cattle comfort.

In the current study, animals were typically surrounding the water trough in all pens, based on anecdotal observation, and drinking was counted both when animals were drinking water and if their head was over the trough. No association was found between the percentage of cattle drinking and treatment in the current study, though previous studies have found that during times of heat events, shade positively impacted behaviors such as eating or drinking (Blaine and Nsahlai 2011; Gaughan et al. 2010; Novelli et al. 2022). Space allowance also had no association with cattle drinking, but an increase of lairage duration was associated with fewer cattle drinking. Since the increase of time in lairage also led to the odds of more cattle lying, it makes sense that there were less cattle observed drinking water.

Motion behavior, such as weight shifting and walking, was scored in the current study. Behaviors such as restlessness, or weight shifting between hooves, has been previously studied in dairy cattle to analyze discomfort (Neveux et al. 2006; Chapinal et al. 2011) and could be an indicator of how cattle settle during lairage. Results indicated that with a unit increase of space allowance or time in lairage there was an increase in the odds of motion decreasing. The current findings suggest that over time cattle settle in the new environment, as Cockram (1990) found to occur after 3.5 h in lairage. There was no impact of shade on motion.

### Dark cutting

Management during the pre-slaughter period can also have negative impacts on meat quality (Scanga et al. 1998; Tarrant et al. 1988; Ferguson and Warner 2008; Romero et al. 2017; Steel et al. 2022). Although the prevalence is relatively low, a common concern of the beef industry is dark cutting beef that occurs due to depletion of glycogen prior to slaughter (Tarrant 1989; Scanga et al. 1998). Dark cutting can not only be an indicator of stress prior to slaughter, it is also an economic concern since dark cutting carcasses are downgraded (USDA-AMS 2017) and have a lower shelf life (Newton and Gill 1981; Ponnampalam et al. 2017). Because the occurrence of dark cutting is multifactorial, it can be difficult to manage. Mitlöhner et al. (2001, 2002) found heifers provided shade in the feedlot had a decrease in dark cutting beef compared to those raised without shade. The literature, however, is limited in understanding how heat mitigation during the pre-slaughter phase impacts this meat quality defect.

The current study found no relationship between shade and the occurrence of dark cutting or off-color carcasses. As dark cutting is generally considered a quality defect associated with more chronic stress conditions, perhaps the brief provision of shade at the slaughter plant was not significant enough to have an impact. In the current study, there was a relationship between THI and dark cutting or off-color frequency; a one-unit increase in THI resulted in the odds of dark cutting occurrence to decrease. This finding is not in line with other studies. Many studies have reported relationships between various weather parameters, including THI, and dark cutting frequency, but results across studies have been variable, and the same environmental measurements are not always utilized. Contrary to the current findings, Scanga et al. (1998) reported that an increase in THI was associated with an increase in dark cutting prevalence. Davis et al. (2024b) reported no association between dark cutting and THI. In a retrospective analysis of historical data, Steel et al. (2022) reported that climatic conditions do have a role in the prevalence of dark cutting but it is a relatively small impact. Additionally, Steel et al. (2022) indicated that THI within the 48 h prior to consignment was not related to dark cutting but that THI between 3 and 28 d prior to consignment was. Additionally, studies have reported that factors such as wind speed, precipitation, and temperature all can impact dark cutting (Murray 1989; Steel et al. 2022; Davis et al. 2024b). Although the shade treatment itself did not have an impact on dark cutting in this study, numerically there was a difference in frequency of off-color carcasses between the two treatments. Future work should further explore how the relationship between the specific metrics associated with the microclimate created in the lairage pen environment interacts with ambient conditions and potentially impact dark cutting and off-color prevalence in fed cattle.

Additionally, space allowance was related to dark cutting in the current study; an increase in space allowance increased the odds of cattle having dark or off-color carcasses. Davis et al. (2024b) reported a unit increase in space allowance was associated with an increase in frequency of dark cutting; that study did not evaluate off-color carcasses, but the impact found was similar to the relationship found in this study. However, Romero et al. (2017) reported a decrease of space allowance was associated with an increase of dark cutting, and Mach et al. (2008) reported no influence of space allowance on dark cutting. Davis et al. (2024b) indicated that it would be valuable to understand cattle behavior during lairage, perhaps providing insight into the relationship between space allowance and dark cutting. In the current study, space allowance was associated with almost all cattle behaviors observed. In this study behavior was assessed at a pen level, and it would be valuable to develop future projects that assess the behavior of an individual animal and how that relates to the incidence of dark cutting. Potentially evaluating how space allowance interacts with other factors (such as distance traveled, time waiting on the truck prior to unload, etc.) would also be important to consider in future work focused on exploring the relationship between dark cutting and space allowance.

Future research should focus on how time of day, behavior during lairage, and past exposure to shade are associated with dark cutting occurrence to gain a better understanding of how short-term environmental experiences impact cattle.

### Survey

Creating a comfortable environment in lairage by providing proper facilities can also improve handler welfare, which in turn directly improves animal welfare. There are numerous studies that have demonstrated the impact that human handlers can have on livestock, both positively and negatively (Cockram and Corley 1991; Grandin 1997; Grandin 2020). In the current study a survey was conducted to understand how the facility employees ­working in the holding area perceived the use of shade. The majority of the survey respondents preferred to work in the shaded pens and agreed shade should be used throughout the facility. While most free response answers indicated that the cattle were easier to move or seemed more calm or “*fresh*” in the shaded pens, some employees also shared that they themselves also enjoyed having the shade. Employees understand the importance of heat mitigation, and previous surveys have shared similar findings (Rusche et al. 2021; Dean et al. 2023). A recent survey, which included feedlot stakeholders (i.e., veterinarians, nutritionists, and owner/operators), explored heat mitigation in feedlots and found that although most of the participants did not utilize shade, most participants believed that providing shade is effective at minimizing heat stress in feedlot cattle (Dean et al. 2023). Similarly, Rusche et al. (2021) reported that 63% of respondents named shade as the most desirable heat stress mitigation in feedlots. Although not a written response, through communication with employees during data collection for the current study, they often talked about not liking the use of sprinklers due to getting them wet or impairing their vision when droplets landed on their safety glasses.

## Conclusion

The provision of shade was not related to mobility, behavior, and dark cutting in the current study. We evaluated one type of shade structure, and future work should be conducted to evaluate different types of shade, exploring different structures, heights, and orientations to maximize potential benefits. Space allowance and lairage duration did have impacts on both behavioral and quality outcomes evaluated. Additionally, shade benefits at plants processing other types of cattle, such as cull cattle (i.e., bulls, dairy cows, etc.) could be beneficial. Employees at the study facility had positive attitudes towards the use of shade in lairage and supported this intervention.

## Abbreviations:

IACUC: Institutional Animal Care and Use Committee
IRB: Institutional Review Board
THI: Temperature humidity index
